# Supplementary material for: Abscisic Acid Improves Insulin Action on Glycemia in Insulin-Deficient Mouse Models of Type 1 Diabetes
Source: Metabolites. 2022 Jun 6;12(6):523. doi: 10.3390/metabo12060523 (PMC9227369; doi:10.3390/metabo12060523)
Supplement: Supplementary file 1 [file metabolites-12-00523-s001.zip › metabolites-1752339-supplementary.pdf]

Supplementary Materials

Figure S1. *Chronic ABA treatment reduces body weight loss without modifying food intake in a multiple low-dose STZ protocol of T1D induction.*

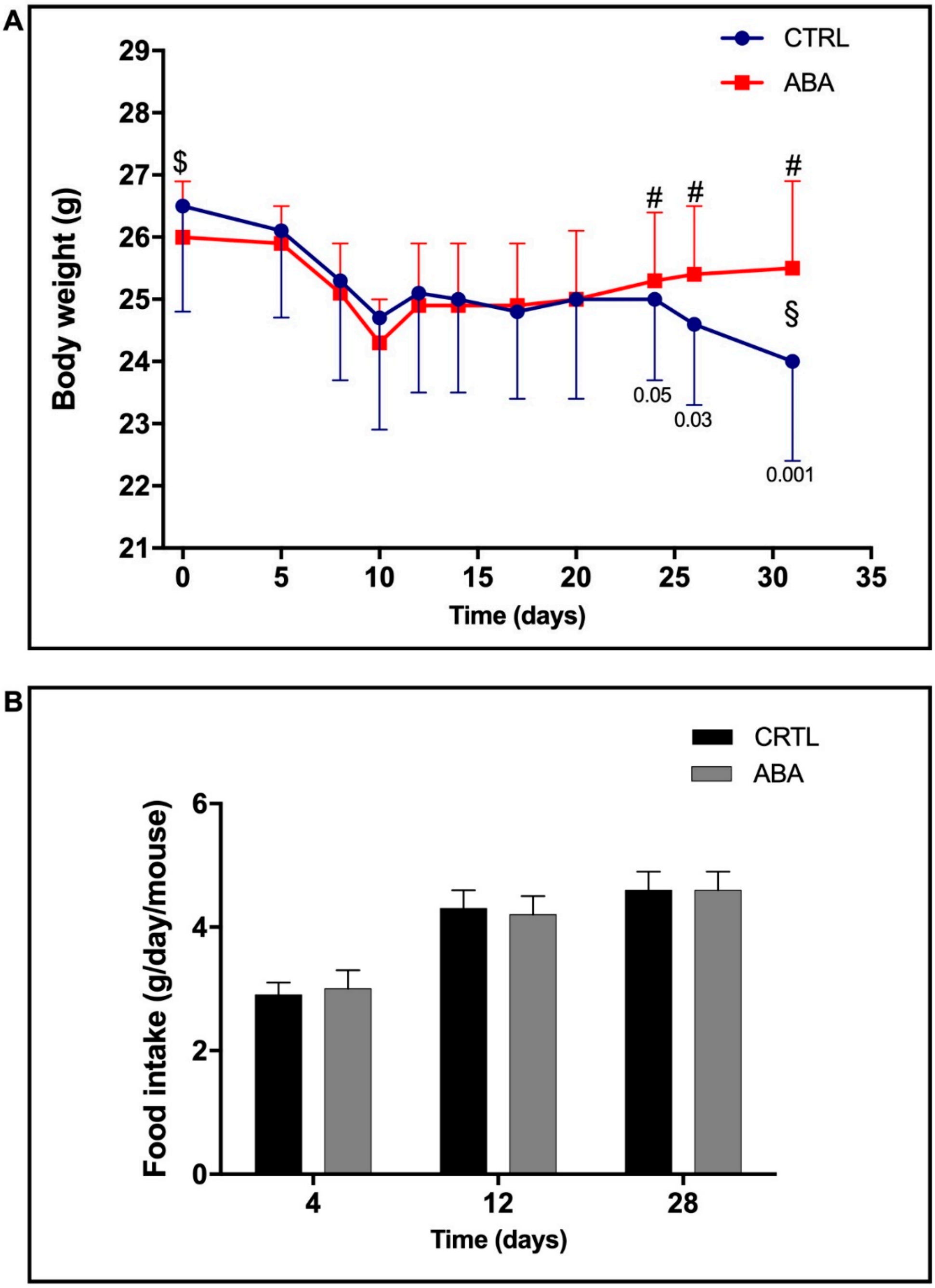

Figure 1S. Eight-week old male LANCL2+/+ (wild-type) mice, obtained from a heterozygous breeding scheme (see Materials and Methods) were divided into two groups (8 mice/group) and treated or not (control, CTRL) with ABA at 5 μg/Kg BW/day starting before diabetes induction with multiple low-dose streptozotocin (STZ) at 20 mg/Kg BW/day for 5 days (see Figure 1A). A. Mice

were fasted for 2 hours before weight measurement. Results shown are the mean ± SD from 8 animals per group. The P values (two-tailed t test) indicated are calculated relative to time = zero in control mice. #, no significant difference between each value and body weight at time = zero in the ABA-treated mice. $, no significant difference between control and ABA-treated mice at time = zero. §, p=0.026 ABA-treated vs. controls. B. Average food intake *per* mouse, *per* day was calculated at the time points indicated in the figure. No significant difference was observed between control and ABA-treated mice at any time point.

Table S1. *Primer sequences used to amplify mouse target genes.*

Mouse genes	Accession Nr.	Forward Primer 5′-3′	Reverse Primer 5′-3′	PCR Product size
Prkaa1	NM_001013367	AGAAGCAGAAGCACGACGG	TTGCCCACCTTCACTTTCCC	93 bp
Slc2a4	NM_009204	CCAGCCTACGCCACCATAG	TTCCAGCAGCAGCAGAGC	161 bp
Ppargc1a	NM_008904	CCCTGCCATTGTTAAGACC	TGCTGCTGTTCCTGTTTTC	160 bp
Ins1/2	NM_008386 NM_001185083	GTGAAGTGGAGGACCCACAA	GCTGGTAGAGGGAGCAGATG	140 bp
IR	J05149	GAGTGCTGCTCATGCCCTAA	GGATGGCCTGGGGACAAAAA	116 bp
Pdha1	NM_008810	GATGGAGCTAAAGGCGGATCA	TCCGTAGGGTTTATGCCAGC	116 bp
GAPDH	GU214026	CGTGCCGCCTGGAGAAACCTG	TGGAAGAGTGGGAGTTGCTGTTGAAG	143 bp
Pfkm	NM_001163487	AGTTGGTATCTTCACGGGCG	CATAGACACGCTCTCCCACG	108 bp
Hprt1	NM_013556	CCCTGGTTAAGCATACAGCCCC	AGTCTGGCCTGTATCCAACACTTCG	89 bp
Lancl2	NM_133737	GCCTCCCTTTCCACCCTAACG	GTCCGCTGTCTTCAGTCCTTCC	110 bp

Table S2. *Primary and secondary antibodies used for Western blot.*

Primary Antibody	Host	Concentrations	Manufacturer
anti-LANCL2	Mouse	1:1000	Reference [8]
anti-PGC-1α	Mouse	1:1000	Sigma-Aldrich (Cat. # ST1202)
anti-AMPK	Rabbit	1:1000	Cell Signaling Technology, Danvers, MA (Cat. # 2532)
anti-GLUT4	Mouse	1:200	Cell Signaling Technology, Danvers, MA (Cat. # 2213)
anti-Vinculin	Rabbit	1:1000	Cell Signaling Technology, Danvers, MA (Cat. # 13901)
**Secondary Antibody**	**Concentrations**		**Manufacturer**
anti-Mouse	1:2000		Santa Cruz Biotechnology Inc., California (Cat. # sc-2005)
anti-Rabbit	1:1000		Santa Cruz Biotechnology Inc., California (Cat. # sc-2004)
